# Minerals from Macroalgae Origin: Health Benefits and Risks for Consumers

**DOI:** 10.3390/md16110400

**Published:** 2018-10-23

**Authors:** Ana R. Circuncisão, Marcelo D. Catarino, Susana M. Cardoso, Artur M. S. Silva

**Affiliations:** Department of Chemistry & Organic Chemistry, Natural Products and Food Stuffs Research Unit (QOPNA), University of Aveiro, Aveiro 3810-193, Portugal; anarcircuncisao@ua.pt (A.R.C.); mcatarino@ua.pt (M.D.C.); artur.silva@ua.pt (A.M.S.S.)

**Keywords:** macroalgae, edible seaweed, mineral profile, toxic metals, functional foods, mineral bioavailability, mineral bioaccessibility

## Abstract

Seaweeds are well-known for their exceptional capacity to accumulate essential minerals and trace elements needed for human nutrition, although their levels are commonly very variable depending on their morphological features, environmental conditions, and geographic location. Despite this variability, accumulation of Mg, and especially Fe, seems to be prevalent in Chlorophyta, while Rhodophyta and Phaeophyta accumulate higher concentrations of Mn and I, respectively. Both red and brown seaweeds also tend to accumulate higher concentrations of Na, K, and Zn than green seaweeds. Their valuable mineral content grants them great potential for application in the food industry as new ingredients for the development of numerous functional food products. Indeed, many studies have already shown that seaweeds can be used as NaCl replacers in common foods while increasing their content in elements that are oftentimes deficient in European population. In turn, high concentrations of some elements, such as I, need to be carefully addressed when evaluating seaweed consumption, since excessive intake of this element was proven to have negative impacts on health. In this regard, studies point out that although very bioaccessible, I bioavailability seems to be low, contrarily to other elements, such as Na, K, and Fe. Another weakness of seaweed consumption is their capacity to accumulate several toxic metals, which can pose some health risks. Therefore, considering the current great expansion of seaweed consumption by the Western population, specific regulations on this subject should be laid down. This review presents an overview of the mineral content of prevalent edible European macroalgae, highlighting the main factors interfering in their accumulation. Furthermore, the impact of using these marine vegetables as functional ingredients or NaCl replacers in foods will be discussed. Finally, the relationship between macroalgae’s toxic metals content and the lack of European legislation to regulate them will be addressed.

## 1. Introduction

Marine macroalgae are currently pointed as the plant-origin foods from the future, earning already the status of “superfoods”, which is a market term for the recognition of their supposed health benefits as a consequence of their superior nutritional profile and richness in bioactive phytochemicals [1,2,3]. They are very low in fat, although they contain high percentages of mono and polyunsaturated fatty acids, and are very rich in carbohydrates (mainly dietary fibers), proteins, containing all the essential amino acids, and vitamins, including vitamins A, C, E, and those of the complex B, which are usually absent in land vegetables [4]. Additionally, the value of edible seaweeds in human nutrition is also based on their richness in several minerals, like sodium (Na), magnesium (Mg), phosphorous (P), potassium (K), iodine (I), iron (Fe), and zinc (Zn) [5]. Because these organisms have strong bioadsortive and bioaccumulative capacities, their mineral content may be 10 to 100 times higher than that of land vegetables. In fact, the ash content of certain seaweeds may reach up to 40 % on a dry weight basis (DW) [6], while only 20% DW have been reported for spinach, which is considered to have an exceptional mineral content [6]. Most algae display higher Na and K values than those reported in vegetables, but usually low Na/K ratios [4], which is an important aspect for good maintenance of cardiovascular health, since low Na/K ratios are well known to promote the decrement of blood pressure [7]. High Na levels, however, may also be regarded as one of the weaknesses of seaweed consumption, considering that in many developing and developed countries, the intake of this element is already above the recommended daily allowances (RDA) [8]. Furthermore, calcium and phosphorus, the two major minerals in the human body, alongside with magnesium, are abundant elements in algae as well, present in concentrations that surpass those of apples, oranges, carrots, and potatoes [9]. This is particularly important considering that, currently, we are witnessing a quick escalation of plant-based dietary movements and lifestyles that limit or exclude the consumption of meat, eggs, and dairy products, which are the major sources of such minerals. In addition, seaweeds, especially species from Phaeophyceae, may accumulate exceptional levels of iodine, which is well known to be an essential element for the maintenance of thyroid function and health. Considering that iodine deficiency is a reality at least in 11 European countries and most of the remaining countries are using iodized salts to control this problem, the introduction of seaweeds in population eating habits could be a valid alternative to ensure intake of the optimal daily requirement of iodine. Nevertheless, the consumption of such seaweeds must have some precautions since the daily intake of more than 600 µg of iodine (tolerable upper intake level for adults) may act in the opposite direction, causing poisoning effects [10].

On the other hand, seaweeds may also accumulate toxic metals (e.g., arsenic (As), cadmium (Cd), copper (Cu), mercury (Hg), and lead (Pb) in varying degrees that might represent up to 200–500 times those of land plants. This fact is one important aspect to have in mind when considering seaweed consumption, since it could represent a potential health risk [9]. Values of toxic metal in the majority of edible macroalgae are, however, usually below the maximum concentrations allowed for human consumption in most countries [11]. Moreover, one must note that the negative effect of the toxic metals depends on their physical state. For example, As is much more toxic in its inorganic than the organic form, the latter being the predominant form found in seaweeds [12]. Hence, even though seaweeds might have high levels of As, it does not necessarily mean that their consumption will cause poisoning effects. Nevertheless, there is currently no legislation in the European Union addressing the limits of toxic elements in edible seaweeds. An exception is made for France, which is the only European country that has already defined limits of potentially toxic compounds in seaweeds to be used for human consumption. Even though, these are only recommendations from the food safety authority and are not legally binding [13].

The nutritional properties and physicochemical composition of seaweeds are, however, very dependent on the algae species [14,15], location [16,17], seasonality [18,19], and cultivation conditions [20,21]. In this context, the aim of this work is, in a first approach, to summarize the major minerals present in red, green, and brown seaweeds from different locations along the European coast and describe their principal accumulation mechanisms. Additionally, it will also address relevant studies focused on the potential of seaweeds to serve as functional ingredient in foods, regarding their mineral profile. Finally, the relationship between seaweed consumption and possible negative health effects due to toxic metals accumulation will also be addressed, highlighting the lack of European legislation.

## 2. Materials and Methods

Two authors (A.R.C. and M.D.C.) collected data from 1989 to 2018 in different databases, including Scopus, Web of Knowledge, Google Scholar, and PubMed, using the keywords “seaweeds OR macroalgae AND mineral assimilation OR mineral uptake OR mineral accumulation”; “edible seaweed OR edible macroalgae AND mineral content OR mineral profile OR elemental profile”; “European seaweeds consumption OR European macroalgae consumption”; “recommended daily allowances AND macro elements OR trace elements”; “seaweeds OR macroalgae AND functional foods OR functional ingredients”; “seaweeds mineral OR seaweeds elements OR macroalgae mineral OR macroalgae elements AND bioavailability OR bioaccessibility”; “edible seaweeds OR edible macroalgae AND heavy metals OR toxic metals”, and “toxic metals OR heavy metals AND tolerable daily intake OR legislation OR recommendations”. The selected studies focused on edible macroalgae from Europe, addressing their mineral uptake and elemental composition either in macro and trace minerals or in toxic metals. Since no legislation on edible macroalgae toxic metals is yet defined, documents stating the tolerable daily intakes and recommendations of these elements were also included to help develop an understanding of the potential risk of their presence in seaweeds. Studies focusing on edible macroalgae as ingredients for improvement of foods’ mineral profile have also been selected, as well as those addressing the bioaccessibility and bioavailability of macro and trace minerals of edible macroalgae. No language restrictions were imposed.

## 3. Accumulation of Minerals by Macroalgae

In general, it is accepted that the accumulation of nutrients in macroalgae can enclose distinct processes, including uptake (i.e., diffusion through cell wall and transport across the cell membrane) and assimilation (i.e., incorporation into cellular components). Still, to the best of our knowledge, very few studies were carried out focusing on the mechanisms of mineral accumulation by macroalgae.

Overall, minerals are taken up from the growth medium towards cytoplasm as charged particles, i.e., ions. However, note that some ions, particularly cations, may not reach the cell membrane due to their adsorption to chemical components of the cell wall. In addition, one cannot forget that the charge difficults diffusion across the cell membrane, which is electrically polarized and has several other components that either repel or attract ions [22]. Hence, unlike non-polar small compounds, elements cross the cell membrane by facilitated diffusion or by active transport, which are processes dependent on factors, such as the ion charge and concentration, as well as the electrochemical potential gradient both inside and outside of the cell.

Accumulation of minerals by macroalgae is believed to depend either on intrinsic (e.g., specific forms of functional groups, such as hydroxyl, carboxyl, amino, and sulfhydryl ester from polysaccharides, proteins, and/or lipids) and external (e.g., pH, temperature, salinity, and interferents in the growth medium) factors. Yet among them, the content and/or type of polysaccharides in the macroalgae cell wall constitute a pivotal determining aspect on the mineral uptake process. In this context, because of their structural and physiological features, brown algae are generally recognized for their superior ability to accumulate minerals [23]. Indeed, polysaccharides in the cell wall of brown seaweeds are mainly composed of alginates and sulfated polysaccharides, which contain numerous polygalacturonic acid units that represent anionic carboxylic binding sites able to interact with metallic cations [24,25]. This fact, together with the presence of vanadium-haloperoxidases in the cell wall of brown macroalgae that promote the oxidation of iodide to hypoiodous acid and molecular iodine, enable the accumulation of iodine to more than 30,000 times over its concentration in the surrounding environment. On the other hand, despite being less effective than the brown macroalgae alginates and sulfated polysaccharides, agar and carrageenan present in the cell wall of red algae also contain hydroxyl and sulfate anionic groups that can establish interactions with cations [26] and enable their accumulation. Likewise, cell walls of green macroalgae, particularly those from the *Ulva* genus, are composed mainly of ulvans, i.e., acidic water-soluble sulfated heteropolysaccharides. As for brown and red algae, these polysaccharides have also ionic binding properties due to their structure composed of sulfate groups linked to rhamnose and xylose residues, as well to carboxyl groups present in glucuronic and iduronic acids [27,28,29]. Indeed, for ulvans from *Ulva rigida*, Paradossi et al. [30] have reported a proportional correlation between the fixation of copper and the content of uronic acids, as well as the participation of sulfates in the binding process for high concentrations of this element.

In addition to the cell wall composition of seaweeds, other physical and chemical parameters (wave exposure, seawater temperature, light, salinity, pH, oxidation state of minerals, etc.) may influence the mineral uptake rates. Note that most of these parameters are seasonality-dependent and affect the chemical composition of macroalgae, hence influencing their mineral and/or nutrient uptake mechanisms. Additionally, macroalgae’s morphological characteristics and life stage must be considered as well. As an example, Nitschke et al. [31] found that the accumulation of iodine in *Saccharina latissima*, *Laminaria digitata*, and *Laminaria hyperborea* varied considerably between different thallus parts, increasing from distal towards basal blades and stipes.

Furthermore, it should be noted that the seaweeds’ elemental analysis has been carried out by distinct methodologies that differ regarding sensitivity and accuracy. Atomic absorption spectroscopy (AAS) is a very trustful methodology and highly sensible for detecting elements but is also very time consuming for routine analysis since it requires the analyses of one element at a time. This technique is being replaced by others, mainly by inductively coupled plasma optical emission spectroscopy (ICP-OES) [32,33,34] and inductively coupled plasma mass spectrometry (ICP-MS) [35,36,37]. The former allows analysis of multiple elements at a time, but, as it lacks sensitivity to detect ultra-trace elements, it is only adequate for macro-elemental analysis. In turn, the latter is a technique that combines the sensitivity of the AAS, with the possibility of multiple elemental analysis of the ICP-OES, but it is very expensive and inadequate for small labs with simple needs [38]. Despite being less frequent, other methodologies, such as non-destructive photon activation analysis [39], neutron activation analysis [40,41], or X-ray fluorescence analysis [42,43], have also been employed for the elemental analysis of seaweeds. Therefore, in addition to the environmental and physiological parameters, the techniques used for assessment of macroalgae’s mineral content must also be considered as a cause of variability.

## 4. Mineral Content of Macroalgae

Table 1 summarizes the mineral content found by different authors in dominant edible seaweeds, mostly of European origin, from the three different phyla. Note, however, that as environmental factors, like seasonality, are not considered, the presented data might only provide mineral levels as a snapshot. Regardless of the high variability found between the studies, it is possible to suggest that, in general, the contents of sodium and potassium in *Ulva* spp. (i.e., the prevalent edible Chlorophyta) tend to be lower than those found in red and brown macroalgae*.* Likewise, particular high amounts of K have been described in red seaweeds, *Gracilaria* spp. and *Palmaria palmata*, collected in Portugal and from an unknown location, as well as for the brown macroalgae, *Laminaria* spp. and *Saccharina latissima*, from Spain and Norway [32,44,45,46]. It is of relevance to note that comparable levels of Na (79 g/kg DW) have been reported for *U. pinnatifida* although this has been collected in Japan [47]. Moreover, the same authors also detected high K levels in the red macroalgae, *Gracilaria* spp. (136–201 g/kg DW) and *P. palmata* (95 g/kg DW), from Hawaii and Maine, respectively. 

In addition to their mineral wealth, macroalgae also stand up for their low Na/K ratio, quite below those found in diverse food products, such as cheddar cheese (8.7), olives (43.6), and sausages (4.9) [6,48]. Overall, data in Table 1 point to an Na/K ratio ranging between 0.9 to 1 for green, 0.1 to 1.8 for red, and 0.3 to 1.5 for brown seaweeds. This ratio was reported to be particularly low in *P. palmata* (0.1) and *Laminaria* spp. (0.3–0.4), both from Spain origins [45,49]. Note that the Na/K ratio recommended by the World Health Organization (WHO) is close to one, so consumption of food products with this proportion or below should be considered for healthy cardiovascular purposes [50]. On the other hand, one must take into consideration that currently most of the developing and developed countries are ingesting a mean level of Na of 3.95 mg/day, which is almost twice the RDA established for this element [8] and, therefore, although seaweeds have an equilibrated Na/K ratio and their consumption could contribute to an increase of K intake, their Na levels are in general high as well, and this fact can result in a global increment of Na intake in the diet, if no salt replacement is considered. By contrast, the use of seaweeds as NaCl replacers in processed foods could represent a good strategy to reduce the overall consumption of Na while increasing, at the same time, the intake of K and other deficient elements that otherwise would not be present in NaCl salted foods. As for Na and K, the ingestion of Ca and Mg are also correlated with cardiovascular health. Indeed, it has been suggested that an appropriate Mg intake may decrease blood pressure, since it acts as a calcium antagonist on smooth muscle tone, thus causing vasorelaxation [51]. The studies reviewed in Table 1 show that green seaweeds tendentially accumulate more Mg than Ca, while the opposite occurs in brown seaweeds. In turn, with the exception of *Phymatolithon calcareum*, which can accumulate particularly high concentrations of Ca [46], red seaweeds generally contain lower, but balanced, concentrations of these two minerals compared to the two other macroalgae groups. Interestingly, particularly high amounts of Ca have been described for *Chondrus crispus* (54 g/kg DW). Such atypical levels may, however, indicate the presence of some calcareous epibionts that are interfering with the analysis [52]. It must be highlighted that the Ca/Mg ratio is also relevant with respect to calcium absorption, since a deficient magnesium intake can result in an excessive accumulation of calcium in soft tissues, thus leading to the formation of kidney stones and appearance of arthritis [53,54]. At last, phosphorus (P) levels seem to be present in similar amounts in the three macroalgae groups, with varying concentrations ranging from 0.5 to 7 g/kg DW (Table 1)*.* Although not represented in Table 1, seaweeds also contain large amounts of sulfate (1–6 % DM), which is typically found in their polysaccharides. These sulfate levels are believed to be closely related to salt concentrations in the water and other specific aspects of ionic regulation [55].

Once again, one must highlight that the high dispersion of data collected for each macroalgae species hampers solid conclusions regarding their specific capacity in accumulating a specific mineral, alerting to the need for comparative studies. Naturally, variations in literature are due to a multitude of factors, including the geographical location, seasonality, human exploratory activities, processing (e.g., type of water in washing procedures), and laboratory manipulations, among others. One such case was reported by Larrea-Marín et al. [45], who found that the Na content of *Porphyra* collected in Spain and France was about 10 times higher than that of Japanese and Korean *Porphyra* samples. The authors attributed this fact to the treatment undergone by *Porphyra* from Asiatic countries, before its selling to the Spanish market for sushi preparation. On the other hand, the recent reclassification of Asiatic *Porphyra* to *Pyropia* may be the real explanation of such differences in Na contents, since they have probable variations on morphological and physiological characteristics.

Among environmental factors, seasonal effects are believed to have a great influence on the mineral profile of seaweeds. For instance, Schiener et al. [56] reported that the concentrations of Na and K in *Laminaria* spp. (23–40 and 27–67 g/kg DW, respectively) and *Saccharina latissima* (23–37 and 47–67 g/kg DW, respective) both from Scotland, more than doubled from summer to the winter season. The same tendency was reported by Adams et al. [57] for the brown macroalgae, *Laminaria digitata*, from the UK, with Na and K contents being raised from 30 g/kg DW and 23 g/kg DW, respectively, in summer to 46 g/kg DW and 59 g/kg DW, respectively, in winter.

In addition to the above mentioned minerals, seaweeds can also be a source of trace elements, such as iron (Fe), manganese (Mn), copper (Cu), zinc (Zn), cobalt (Co), molybdenum (Mo), selenium (Se), and iodine (I), which are essential for health maintenance. As known, Fe acts as a component of multiple metabolic processes, including oxygen transport, electron transfer, and oxidase activities, while Mn is a cofactor of many metalloenzymes (e.g., superoxide dismutase, arginase, and pyruvate carboxylase) and is associated to amino acid, lipid, and carbohydrate metabolism [58]. Likewise, Cu and Zn are central components of many enzymes, including those involved in neurotransmitter synthesis, energy metabolism, and collagen/elastin cross-linking [59]. Moreover, Co is required for the synthesis of vitamin B_12_, which in turn, is a key coenzyme in propionate metabolism and in cytosolic transmethylation of homocysteine. Furthermore, Mo is essential to the activity of certain enzymes (e.g., sulfite oxidase and xanthine oxidoreductase) that catalyze redox reactions, while Se is mainly present as part of selenoproteins, which have a variety of functions, including antioxidant effects, T-cell immunity, thyroid hormone metabolism, and skeletal and cardiac muscle metabolism [5]. Instead, iodine is vital for normal growth and development, since it is needed to produce thyroid hormones [10]. The reported contents of these elements in European edible macroalgae are also summarized in Table 1.

Notably, Fe is abundant among the three macroalgae groups, although some prevalence occurs in Chlorophyta in comparison with Rhodophyta and Phaeophyta. However, note that, although in lower concentrations, species from the later phylum (e.g., *Alaria esculenta*, *Saccharina latissima*, and *Fucus* spp.) can also be suggested as a good supplier of Fe, as accumulation in some cases can rise above 1 g/kg DW [56,60,61]. The predominance of Fe in *Ulva* spp. with respect to red macroalgae (*Porphyra* spp.) was also confirmed by Astorga et al. [62] in samples from Chile. Albeit that, one must recall that significant variations can occur among the same species. For instance, Dawczynski et al. [63] reported that the Fe content of *Pyropia* from China (991 mg/kg fresh matter, FM) was about seven times higher in specimens from Japan and Korea. On the other hand, the same authors highlighted that Fe contents in brown macroalgae were much dependent on the species, with levels in *Sargassum fusiformis* reaching about three to four times those of *Laminaria* sp. and *Undaria pinnatifida*.

In turn, the highest contents of Mn were reported to occur in red seaweeds, particularly in *Chondrus crispus, Palmaria palmata,* and *Gracilaria* spp., all collected in different regions of Denmark [52]. The preferential accumulation of Mn by red over brown macroalgae was also described by Dawczynski et al. [63], in samples from China, Japan, and Korea. The same tendency was observed by Netten et al. [64], who found the highest Mn content in red macroalgae, *Pyropia tenera*, (32–49 mg/kg DW) from different cities of Japan, with this content being higher than those documented in Table 1 for *Porphyra* spp. from different European countries. Cu in European green macroalgae, *Ulva* spp., was reported to vary between 2 to 33 mg/kg DW, with maximum levels corresponding to samples from Portugal and Ireland, whereas its amounts in red and brown macroalgae ranged from 1 to 35 and 0.3 to 80 mg/kg DW, respectively.

High variable amounts of Zn have also been found by authors, even for the same macroalgae species. For instance, Wallenstein et al. [60] reported a Zn content in *Fucus spiralis* from four different locations in São Miguel Island (Portugal) ranging between 15 to 740 mg/kg DW. Still, the overall reported data suggests that the accumulation of this element in macroalgae is prevalent in brown and red macroalgae, in detriment of Chlorophyta. Among the first, *Fucus* were reported to accumulate up to 740 mg/kg DW, while the ranges of 9 to 74 mg/kg DW, 10 to 82 mg/kg DW, and 24 to 163 mg/kg DW were described to occur in the red macroalgae *Chondrus crispus*, *Porphyra* spp., and *Gracilaria* spp., respectively. In turn, levels in *Ulva* species from distinct European locations did not exceed 64 mg/kg DW. Notably, the levels of this element found by Dawczynski et al. [63] in brown macroalgae (10–33 mg/kg DW) and in *Pyropia* (37 mg/kg DW) from Japan, China, and Korea were lower than those of European samples. The same tendency was observed by Netten et al. [64] in red *Pyropia tenera* (31–37 mg/kg DW) from Japan, brown *Fucus vesiculosus* (24 mg/kg DW) from Norway, and *Undaria pinnatifida* (14 mg/kg DW) from Japan.

Data summarized in Table 1 also allow the conclusion that, in general, Co and Mo tend to mainly accumulate in Phaeophyta. Noteworthy, the maximum levels of Co in European samples were reported for *Ascophyllum nodosum* from Norway [32], while levels of Mo were shown to be more relevant in the brown algae, *Alaria esculenta* and *Saccharina latissima*. Instead, Se seems to be well distributed among the three classes, ranging between 0.03 to 2, 0.1 to 1, and 0.02 to 1.3 mg/kg DW in edible Chlorophyceae, Rhodophyceae, and Phaeophyceae, respectively. Outside Europe, Netten et al. [64] found higher Se levels in brown macroalgae from Japan, particularly in *S. latissima* (6 mg/kg DW) and *Sargassum fusiformis* (4 mg/kg DW) comparatively to red one, *Pyropia tenera* (2 mg/kg DW), with these levels being higher than those reported for Europe. Still, surprisingly, Smith et al. [83] reported superior Se contents in the red *Porphyra* spp. (0.2 mg/kg DW) than those of the brown *U. pinnatifida* (0.07 mg/kg DW), in samples from New Zealand. 

Seaweeds, particularly those belonging to Phaeophyceae, are well-known for their capacity to accumulate exceptionally high concentrations of iodine. Indeed, levels of I on these organisms may reach concentrations that cannot be found in any edible land plant [84]. Major contents of iodine were documented in *Laminaria* spp. and *S. latissima,* both from Scotland origins [56]. Indeed, as previously described by Küpper et al. [85]*,* Laminariales have a particular affinity to accumulate iodine, mainly as iodide and only in small amounts as iodate. Naturally, one must consider that iodine concentrations also vary greatly among species, as well as with growth conditions, development, geographical habitat, and harvest conditions, among other factors [5,56]*.* In this way, Schiener et al. [56] reported higher I contents in *L. hyperborea* (6685 and 10,239 mg/kg DW) and *L. digitata* (9014 and 8122 mg/kg DW) during Spring and Autumn, respectively, with these contents being much higher than those observed for *A. esculenta* and *S. latissima* in the same seasons. Red seaweeds also have been shown to contain considerable levels of iodine, particularly, in *P. palmata* from Norway that showed values of about 260 mg/kg DW [71]. Once again, regarding North Europe, the quantity of I in *P. palmata* from Iceland and Denmark was shown to vary between 5 to 7 mg/kg DW, suggesting that geographical location, as well as environmental parameters, actually have a great effect on elemental content in seaweeds [86]. Note, however, that the excess intake of this element may result in poisoning effects that have been linked to the onset of thyroid cancer, nodular goiter, hyperthyroidism, or hypothyroidism, especially in populations that are considered to follow iodine-sufficient diets [85,86]. Indeed, it has been demonstrated that the consumption of high quantities of *Saccharina japonica*, with consequent iodine concentrations above the tolerable upper intake level (600 μg for adults), by Japanese people (considered to have iodine-sufficient diets) significantly deregulated the serum profile of thyroid hormones, including serum thyrotropin, free thyroxine, and free triiodothyronine, on test subjects, although the values normalized shortly after the subjects returned to their usual dietary habits [87,88]. Interestingly, even in populations considered iodine-deficient, small increases in iodine intake may also change the pattern of thyroid diseases. This is because when dietary iodine intakes are deficient, thyroid accumulates an increased proportion of ingested iodine, reuses the iodine from the degradation of thyroid hormones more efficiently, and renal clearance of iodine is reduced [89]. Therefore, some precautions must be taken regarding the consumption of iodine-rich seaweeds although, despite the increasing consumer acceptance, seaweeds are still regarded as an exotic food item in Europe and their consumption is not enough to exceed the recommended levels. In fact, the inclusion of seaweeds in the diet could support current efforts to improve I status among European populations [13].

## 5. Consumption of Macroalgae

The regular consumption of macroalgae in countries from East Asia (mostly in Japan, China, and Korea) has been associated to distinct health benefits, including cardioprotective, neuroprotective, and anti-inflammatory effects [1,3,90]. Indeed, for the past three decades, Japanese have had the longest life expectancy in the world, due not only to favorable economy and health systems, but also to their healthy food habits based on the Okinawan diet, where seaweeds play a great importance [91]. These facts are boosting the Western culture to increase interest in the manufacturing and consumption of macroalgae-derived products. Naturally, the introduction of macroalgae in Western diets may impact mineral intake by populations.

### 5.1. Contribution for the Dietary Mineral Intake

The recommended daily allowance (RDA) for minerals is essential to understand possible benefits and risks resulting from the direct consumption of macroalgae. Table 2 and Table 3 summarize the contribution of different species to RDAs (macro- and trace elements, respectively), using the approach suggested by MacArtain et al. [4], i.e., taking into consideration the average and the maximum mineral content of seaweeds (apart of location) present in an 8 g dry-weight portion, which is a typical daily portion consumed in Asian cuisine. Please note that RDAs were established for nutrition labelling of foodstuffs according to the Commission Directive 2008/100/EC [92] and the WHO guideline [93]. The previous directive also recognized that minerals can only be declared in case they represent a “significant amount”, which corresponds to a minimum of 15% of the RDA supplied by 100 g or 100 mL (or per package, if it only contains a single portion). As shown in Table 2, brown seaweeds are, on average, major Na and K contributors, making them good candidates as salt replacers in food formulations. Additionally, red seaweeds, such as *Chondrus crispus* and *Phymatolithon calcareum*, are the major calcium contributors to RDA [46,52], while the green seaweed, *Ulva* spp., can be highlighted by its Mg richness [44].

Notably, *Ulva* spp. can also be regarded as a great source of Fe and Mn, with a single dose accounting for a mean value of 78% and 49% RDAs, respectively (Table 3). Still, note that, in general, the red macroalgae, particularly *C. crispus, P. calcareum,* and *Gracilaria* spp., can even exceed by far this Mn contribution, with a mean single dose representing ≥70% of the RDA [46,52]. Moreover, all seaweeds species are rich RDA contributors of iodine, with maximum values being found for *Laminaria* genus, for which a dose of 8 g can represent up to about 48,000% of the RDA [56]. In turn, they represent a modest RDA diet contributor of Cu, Zn, Mo, and Se. Among the reported data for European macroalgae, maximum average levels of Cu were found in *A. nodosum* [78] and *F. vesiculosus* [61], while those of Zn, Mo, and Se were reported to occur in *Fucus spiralis* [60], *Alaria esculenta* [32], and *Saccharina latissima* [35], respectively.

### 5.2. Applications as Food Ingredient

Because seaweeds have such an abundant mineral content, many authors have claimed that their consumption would strongly contribute for meeting the recommended daily intake of some essential minerals and trace elements that are usually absent in regular diets [71,94,95]. However, considering that seaweeds have generally high amounts of Na, and that in Western diets, the current intake of this element is already above the recommended levels, even though their consumption could help to increase the intake of other minerals, it would also contribute to an even higher sodium intake, consequently entailing higher health risks. This characteristic may, however, be advantageous if considering seaweeds as a salt replacer in processed foods, since their high mineral content would contribute to the maintenance of foods’ salty taste without being necessary to add NaCl. In this context, many authors have taken advantage of this seaweeds’ feature for reducing the use of NaCl in foods, while enhancing their content in certain elements, such as calcium potassium or iodine, which are usually lacking or below recommended levels in regular diets (Table 4).

The formulation of seaweed-fortified foods aiming to reduce the use of NaCl and improve their mineral content has been particularly focused on meat-based products. In this field, López-López et al. [96,97,98,99] have done remarkable work in the reformulation of several meat products, managing to partially replace the addition of sodium chloride with different species of edible seaweeds, while maintaining their textural and sensory characteristics. Among their different formulations, this research group created meat emulsions, meat patties, and frankfurters enriched with *Undaria pinnatifida*, *Himanthalia elongata*, or *Porphyra umbilicalis*, that were both low in Na and rich in K, presenting Na/K ratios bellow 1, which is much lower than the ratios above 3 found in their conventional recipes [100,101,102]. In addition to the increment of K, low-salt beef patties fortified with 3% *Undaria pinnatifida* showed six to seven times more Ca and three times more Mg than the control [101], while the addition of 5.6% of this seaweed to meat emulsions caused these two minerals to increase 13 and four times, respectively. Moreover, the latter product was found to contain trace amounts of Mn, which was absent in the control samples [102]. Likewise, the content of Ca, Mg, and Mn in different meat products incorporated with *Himanthalia elongata* have been reported to increase up to >1000, >300, and >700%, respectively [100,102], and that of Fe was increased four times in *Porphyra umbilicalis* fortified-meat emulsions [102]. Note that as meat is not a good source of Mg, the formulation of these seaweed-fortified meat products might represent a good strategy to improve the general intake of this microelement [103]. In the field of fish-based products, Hanjabam et al. [33] showed that the incorporation of *Sargassum swartzii* (0–5%) into tuna jerkies resulted in a dose-dependent increase in the levels of several macro- and micro-minerals, namely K, Ca, Mg, Mn, and Fe.

Alternatively, enhancement of the mineral content in meat, fish, and other animal-derived products can be achieved through feeding animals with diets supplemented with algae. Indeed, the supplementation of laying hens with *Ulva prolifera* and *Cladophora* sp. enriched with trace elements (Cu, Zn, Co, Mn, and Cr) was shown to significantly increase the content of Cu, Mn, and Cr in the eggs, alongside other elements, such as K (in the groups supplemented with Cr-, Mn-, and Co-enriched algae), Ca (in all groups except those supplemented with Cr- and Zn-enriched algae), and Mg (in all groups except those supplemented with Zn-enriched algae) [104]. Likewise, He et al. [105] found that, compared with pigs under a normal diet, those supplemented with *Laminaria digitata* over three months accumulated 45% more I in muscle tissue and up to 213% in other internal organs. Similar results were further described in an identical study in which pigs fed with *A. nodosum* contained 2.7 to 6.8 more I in their muscle and internal organs than those fed under a regular diet [106].

Likewise, supplementation of fish with seaweed-fortified meals has proven to be an effective way of improving their fillet iodine content. According to Valente et al. [107], after three months of feeding rainbow trout (*Oncorhynchus mykiss*) with *Gracilaria vermiculophylla*-enriched fish meals (5%), the fish doubled their flesh iodine content compared to the control group. Similarly, feeding chars (*Salvelinus* sp.) with *Laminaria digitata*-fortified fish meal (0.8%) during nine months, caused their total iodine content to be approximately four times higher than that of the control group [108]. Feeding other species, such as gilthead seabream (*Sparus aurata*) and rainbow trout, with *L. digitata*-supplemented meals was shown to positively contribute to the increase of their fillets’ iodine content as well [106,109]. These results are of particular importance considering that aquaculture is overcoming the capture fishery supplies and the current trend in aquafeeds for replacing marine-derived ingredients (fish meal and fish oil) by vegetable protein and oil sources (much lower in iodine) can significantly interfere with the fish nutritional profile.

Milk, dairies, and, more recently, plant “milks” (e.g., soy, almond, oat, and rice) are another segment of food products that occupy a highly significant position in the dietary routines of particular geographical areas of the world, and, therefore, are good candidates for supplementation with macroalgae. In fact, patents for dairy products and plant beverages fortified with seaweed-derived minerals have already been registered [110,111]. Moreover, Aquamin^®^, a multimineral complex from *Lithothamnium muelleri* developed by the Irish firm, Marigot, is currently being used for mineral supplementation of milk, dairies, plant-based “milks”, and even pasta, bakery goods, or snacks [112]. Additionally, some studies focusing on the supplementation of dairies with seaweeds have been carried out. Lalic and Berkovic [113] described that the incorporation of *Undaria pinnatifida* or *Saccharina japonica* of up to 15% in cottage cheeses resulted in higher contents of Ca, Fe, and Mg, although the textural quality was best for cheeses containing only 9% of seaweed. Different concentrations of *Laminaria* spp. have also been incorporated in a new probiotic yogurt, aiming to increase its iodine content. Indeed, contrarily to the conventional yogurt, the fortified formulation contained high levels of I (average of 570 μg I/100 g), as well as considerably incremented amounts of Ca, K, Na, Mg, and Fe [114]. Likewise, addition of *Saccharina latissima* to quark and fresh cheese were shown to act as a source of I and were likely to be accepted by the consumer [115,116]. In the same way that meat, meat products, and fish mineral content may be enriched through livestock feeding, Rey-Crespo et al. [94] found that by incorporating *Ulva rigida*, *Sargassum muticum*, and *Saccorhiza polyschides* as a mineral supplement into cattle forages, the I concentration in milk was doubled compared to the control animals (290 vs. 136 μg/L, respectively). Notably, iodine supplementation through algae-based feeding seemed to be more effective than conventional supplementation with inorganic salts.

### 5.3. Bioaccessability and Bioavailability

The knowledge about the mineral content of seaweeds and/or of their related foodstuffs is not, however, enough to ensure their consumption will cover these elements’ RDAs and provide their known health effects, since there are many aspects that may interfere with their bioavailability in humans. In general, food undergoes different processes after ingestion that can change the type of compound that finally reaches the systemic circulation. In this way, the bioavailability of food nutrients, including that of minerals, greatly depends on the physical properties and physicochemical composition of the food matrix, and the occurrence of different interactions between food components [121]. According to each type of compounds and food matrices, the methodology for the estimation of bioavailability can differ. Up to now, in vitro methodologies are preferable given their simple, rapid, and low-cost advantages, in contrast to the high-cost and ethical problems often associated to in vivo assays. Note, however, that in vitro approaches are mainly designed to evaluate bioaccessibility [122,123], i.e., the amount of compound released from the food matrix during the digestive process that is available to be absorbed. In this context, Afonso et al. [37] recently studied the composition and the bioaccessibility of several elements of different green seaweeds, showing that a higher content of a certain element does not necessarily correspond to its higher bioaccessibility. Indeed, according to their observations, although *Rhizoclonium riparium*, *Ulva prolifera*, and *Ulva lactuca* had identical Zn contents (59.3–63.8 mg/kg dry weight), the bioaccessibility from the former was much higher than the others (64% against 30 and 13%, respectively). On the other hand, despite *R. riparium* showing the highest I content, and *Ulva intestinalis* the lowest (281.5 and 45.1 mg/kg DW, respectively), the bioaccessibility of this element was actually very similar between each species (31 and 29%, respectively). Likewise, Mn bioaccessibility was higher in *U. prolifera* (95%) than in *R. riparium* (80%), even though the latter presented higher concentrations of this element compared to the former (135.1 against 102.1 mg/kg DW, respectively).

Since being bioaccessible does not necessarily mean that minerals will be bioavailable, i.e., absorbed and reaching the systemic circulation, several authors have also focused their studies on the bioavailability of seaweeds’ minerals. In this context, one must highlight that elements are very prone to interacting with dietary fiber, a fact that can compromise their bioavailability because fibers are not accessible for absorption [124]. According to Urbano and Goñi [125], rats fed with *Pyropia tenera* and specially *U. pinnatifida* showed much lower retention coefficients for Ca, Mg, Zn, Fe, Na, and K than the control group, which was attributed to the possible interactions between minerals and seaweeds’ dietary fiber. Indeed, it has been shown that interactions with several anionic polysaccharides, particularly alginates and agar or carrageenan, may form insoluble complexes with minerals, thus decreasing their bioavailability [126]. Nevertheless, studies carried out on growing rats fed with *P. tenera* and *L. digitata* revealed that the apparent absorption values of Na and K were significantly higher compared to their control counterparts (under a regular diet), while Mg bioavailability did not seem to be affected by the consumption of any of the seaweeds tested, thus suggesting low fiber interference in the bioavailability process [127]. Identical findings were observed regarding the bioavailability of these three elements in hypercholesterolemic growing Wistar rats fed with the same algae species [128]. This could be explained by the fermentation that these dietary fibers undergo during their course through the cecum and colon [126], which may promote the release of those retained cations, and thus globally increase their bioavailability.

Notably, some authors further estimated the influence of seaweed consumption on iron absorption. Evaluation of *Kappaphycus alvarezzi* Fe bioavailability through in vitro methods showed that higher absorption efficiency was achieved in intestinal conditions rather than in stomach conditions. Moreover, in the experiments where ascorbic acid was added, a positive correlation between the ascorbic acid concentration and Fe availability was observed [129]. Likewise, in vitro evaluation of Fe bioavailability in *Ulva compressa* and in *U. compressa*-fortified Pakoda revealed that the highest absorption rates (55–56%) occur in intestinal conditions for both products, but the absorption in stomach conditions increases from 15% in the seaweed to 27% in the seaweed-incorporated Pakoda, suggesting that the interactions between food matrices stimulate the absorption of this element [130]. In turn, in an in vitro digestion model of Caco-2 cells, Flores et al. [47] reported that among 13 macroalgae species that included *Ulva* spp., *Palmaria palmata*, *Gracilaria salicornia*, *Gracilaria parvispora*, *Sargassum fusiformis*, *Codium edule*, *Porphyra yezoensis*, *Pyropia vietnamensis*, *Gracilaria coronopifolia*, *Ascophyllum nodosum*, *Ulva lactuca*, and *Undaria pinnatifida*, only *P. palmata* and *U. lactuca* showed higher Fe bioavailability than that of spinach (three and five times higher, respectively), even though six of them had superior contents of this mineral. Improvement of Fe bioavailability was further achieved with the combination of seaweeds with ascorbic acid [47]. Moreover, in a human trial, Garcia-Casal et al. [131] found that *Ulva* sp., *Sargassum* sp., *Porphyra* sp., and *Gracilariopsis* sp. were all good sources of ascorbic acid and of bioavailable Fe, showing similar Fe absorption percentages between each other, either cooked (19.8, 19.8, and 13.9%, respectively) or raw (14.8, 12.2, and 11.3%, respectively). These results suggest that consumption of seaweeds could improve the Fe status of the anemic population and/or the Fe nutrition in people following diets with lower iron disposal, such as veganism. Indeed, the incorporation of *Ulva reticulata* in chocolate not only significantly increased the iron content in the chocolate, but also the Fe bioavailability from 10% in the plain chocolate to 21% in the seaweed-incorporated chocolate. In addition, the seaweed-incorporated chocolate significantly increased the hemoglobin, total iron binding capacity, mean corpuscular hemoglobin and volume, serum iron, and serum ferritin levels in adolescent anemic girls [132].

As the most abundant mineral in the human body, seaweeds’ calcium bioavailability has also been a target of several investigations. According to Bocanegra et al. [127], apparent absorption of Ca in rats fed with *P. tenera* (red) increased 15% compared to their control counterparts, while those fed with *L. digitata* showed a decrease of 10%, even though the latter group ingested the highest amount of Ca (115 mg/day against 93 and 105 mg/day for control and *P. tenera*-fed groups, respectively). This outcome is actually expected since, as a brown seaweed, *L. digitata* is abundant in alginates, which form the well-known egg-box that retain divalent cations, such as Ca [133], and probably hamper the bioavailability of this element. On the other hand, the bioavailability of the calcium supplement, AAA Ca (active absorbable algae calcium, made by heating a mixture of oyster shell and the seaweed, *Sargassum fusiformis*, under reduced pressure), was reported to be exceptionally high. After the oral intake of this supplement, the urinary calcium excretion of the test subjects was 249% of the baseline level, which was significantly higher than that of calcium carbonate (170% of the baseline level), suggesting that the Ca bioavailability of this seaweed-derived supplement is much higher than that of calcium carbonate in humans [134].

Although seaweeds are usually recognized as excellent sources of I, it does not necessarily mean that it will all be bioaccessible and bioavailable upon ingestion. In fact, a relevant in vitro study on I bioavailability encompassing nine different types of seaweed, including *P. palmata*, *Porphyra* spp., *Laminaria* spp., *U. pinnatifida*, *H. elongata*, and *U. rigida*, demonstrated that the I dialysability percentage in the majority of the tested samples was below 5%, except for *Laminaria* spp. and *P. palmata*, which showed 17 and 10% I dialysability, respectively, suggesting that the bioavailability of this element is actually reasonably low [65]. Supporting this data, Domínguez-Gonzaléz et al. [135] later found in a similar study that although the bioaccessibility of I from *Undaria pinnatifida*, *Sargassum fusiformis*, and *Saccharina japonica* after gastrointestinal digestion was considerably high (49–82%), less than 28% of the initial content was bioavailable upon in vitro assay using the dialysis membranes. Moreover, this value was reduced to a mere <3% when the bioavailability analysis was performed in a biological system model consisting of two major cell types present in the intestine, namely absorptive (Caco-2 cells) and mucus-secreting cells (HT29-MTX). These authors have also shown that di-iodotyrosine, i.e., one of the organic forms of I commonly found in seaweeds, has very low permeability in Caco-2 cells, which suggests its poor absorbability (<20%) [135]. Additionally, possible protein interactions (e.g., pepsin, pancreatin, or biliary extract) may also affect the bioavailability of this I form [136]. Nevertheless, absorption of inorganic I, which is the predominant form in brown seaweeds, was observed to be moderate (20−70%). Therefore, the low bioavailability observed for I may be related to the interaction of iodine with other compounds present in the seaweed matrix, forming soluble complexes with greater molecular weight [135]. In vivo trials carried out with iodine-insufficient women supplemented with encapsulated *Ascophyllum nodosum* have, however, revealed more favorable results, since 33% of the ingested I was found to be bioavailable. Even so, this percentage is still low when compared to the control group fed with KI (59%), currently used in iodized salts [137]. 

In vitro estimation of the bioavailability of different trace elements, namely Cr, Co, Mn, and vanadium (V), from several seaweeds revealed that Mn was the element with the highest percentages of dialysability in all the studied samples (>60%). From the total content found in seaweeds, Co and Cr dialysability varied between 33–78% and 30–67%, respectively, showing the best results for *U. pinnatifida*, while the highest dialysability of V was found for *P. palmata* (61%) [135]. Absolute absorption of Mn was, however, not significantly affected in Wistar rats fed with 7% of *P. tenera* or *L. digitata* [127]*,* and even lower in rats under a hypercholesterolemic diet supplemented with the same amount of these two species [128]. Also, in *P. tenera* and *L. digitata*-supplemented Wistar rats, an increased absorption of Cu was seen in animals under a normal diet [127], but not under a hypercholesterolemic diet [128]. In turn, an increased absorption of Zn was only observed in normal dieting-rats supplemented with *P. tenera* [127]*,* but the opposite occurred in rats following an hypercholesterolemic diet, i.e., supplementation with this algae actually caused a reduction of the Zn absorption [128]. Therefore, it is evident that in addition the possible interactions occurring between minerals and seaweeds’ matrices, the bioavailability of seaweeds’ minerals may also be affected by other co-ingested foods as well as different dietary patterns. Indeed, Raes et al. [138] reported that several components in food matrices can also exhibit retention properties in minerals, such as phenolic compounds and phytic acid, which have both shown to reduce the bioavailability of Fe and Zn.

In this way, further studies are necessary to better comprehend how different variables, such as geographic locations, cultivation conditions, processing, co-ingestion, and/or incorporation in other foods, could affect the bioavailability of minerals from macroalgae.

## 6. Worries Related to Macroalgae Consumption

Regardless of being good sources of minerals, macroalgae can also accumulate undesirable compounds, namely, metalloids, like arsenic and antimony (Sb), and toxic metals, such as cadmium, lead, mercury, tin (Sn), strontium (Sr), and aluminum (Al), which can represent a health risk for consumers. The accumulation of such contaminants is naturally also influenced by many environmental factors (e.g., salinity, temperature, pH, light, and nitrogen availability), as well by the structural features of the algae [31,66]. Indeed, distinct authors reported the accumulation of metals as a function of their concentration in the surrounding water and the uptake capacity of seaweeds [139]. It is accepted that this latter process occurs in two ways, either involving (1) a surface reaction, in which metals are adsorbed by algal surfaces through electrostatic attraction to negative sites, or, alternatively, (2) a slower active uptake, in which metal ions are transported across the membrane into the cytoplasm. While the first reaction is mainly influenced by the concentration of minerals and other metals in the surrounding water, and to a lower extent by factors, such as temperature, light, pH, and age of the plant, the second mechanism is mostly dependent of metabolic processes (these are also susceptible to changes of the above described factors). Note that the chemical nature of each element and the conditions that can affect its abundance and speciation are also relevant factors to take into account, since several studies entirely consider metals as divalent cations, which will not be true in many cases [140].

Having in mind the serious environmental problems raised in coastal areas by urbanization and industrialization, the concentration of toxic elements in edible macroalgae is now a growing concern, mainly considering their increment in Western diet. One must highlight that in Europe, seaweeds are commonly considered as “novel foods” and France was the first country to lay down regulations on the use of seaweeds for human consumption as non-traditional food substances. Overall, 21 species of macroalgae and three species of microalgae are authorized as vegetables and condiments in Europe [141]. The maximum levels of toxic metals stipulated by France for edible seaweeds are Pb < 5 mg/kg DW, iAs < 3 mg/kg DW, Cd < 0.5 mg/kg DW, Hg < 0.1 mg/kg DW, and Sn < 5 mg/kg DW [141]. In addition to France, neither Spain nor the other European countries have specific regulations regarding the limits of toxic metals in seaweeds for human consumption. In these countries, the only existing legislation concerns foodstuffs, where the European Commission Regulation (EC No 629/2008) have set the maximum levels of Cd < 3 mg/kg DW in food supplements whose composition is exclusively or mainly composed of dried seaweed or seaweed-derived products [142].This same entity (EC No. 1275/2013) also set the maximum arsenic levels in complementary feed and/or a complete feed meal at 40 and 10 mg/kg (moisture content of 12%), respectively [143]. The absence of specific regulations regarding Cd, Pb, and Hg in foodstuffs containing seaweeds or derived products impelled the “Industrial Contaminants” working group (belonging to the European Commission) to decide whether general advice or a special regulation should be issued [144]. Unexpectedly, in Asian countries, which are responsible for most seaweeds’ production and consumption as human food, no specific limits have been established regarding its toxic metals content. Likewise, the USA and the countries belonging to South America have not regulated the content of toxic metals present in seaweeds. Still, even though no maximum limits were established for arsenic, the Food and Drug Administration (FDA) has monitored As in foods for decades along with the Environmental Protection Agency (EPA) [145].

### Contents of Toxic Metals and Potential Risks

As and Sb represent one of the main concerns of contaminants in macroalgae. These two have a similar chemistry and toxicity, even though Sb is much less abundant in nature than As [146]. The latter may occur in four oxidation states, namely as As(V), As(III), As(0), and As(-III), which can be present in organic or inorganic forms. In macroalgae, organic As appears mostly as arsenosugars and, sometimes, as methyl derivatives [monomethylarsonic acid (MMA), dimethylarsinic acid (DMA), and arsenobetaine (AsB)], while the inorganic forms are mainly represented by arsenate (AsV) and arsenite (AsIII). In general, organic forms exert low or no toxicity [147,148], while the inorganic are undoubtedly the most hazardous, with their accumulation being already associated to the development of several disorders, including nephrotoxicity, diabetes, hepatotoxicity, cardiovascular dysfunction, and cancer, mainly at the skin, lungs, and bladder level [149,150,151,152].

Table 5 summarizes the contents of total As and inorganic As (iAs) found by distinct authors in relevant edible macroalgae, mostly of European origin. Overall, the highest levels of As were found in brown seaweeds, while its amount in red and green macroalgae varied from 1 to 50 and 0.8 to 15 mg/kg DW, respectively. Interestingly, studies conducted by Almela et al. [153] and Besada et al. [72] found that the amount of As accumulated in seaweeds seems to be more dependent on their phylogenetics than on its abundancy in surrounding media. Amongst the analysed macroalgae found in different world locations, *Sargassum fusiformis* was reported to contain the maximum content of As and iAs, followed by *Laminaria* spp. and *Saccharina latissima*. The predominance of this element in brown macroalgae with respect to Chlorophyta and Rhodophyta was also observed by Netten et al. [64] and Dawczynski et al. [63]*,* who reported As levels of 88 mg/kg DW and 87.7 mg/kg FW, respectively, for Japanese *S. fusiformis*. Similarly, no significant differences were found by Ródenas et al. [38] regarding the As levels of European and Asiatic samples of *Laminaria* and *Porphyra/Pyropia*.

Considering the French legislation as a reference, one can conclude that from the data summarized in Table 5, only *S. fusiformis* and *Laminaria* spp. exceeded the 3 mg/kg DW limit established for iAs. Note still that in such cases (85 mg/kg DW for *S. fusiformis* and 20 mg/kg DW for *Laminaria* spp.), the daily consumption of a typical Asiatic portion of algae (8 g) would correspond to 466 and 110% of the Tolerable Daily Intake (TDI) established by the WHO (0.146 mg of iAs/day for a body weight of 68 kg), respectively. Indeed, the consumption of brown seaweeds, particularly of *S. fusiformis*, should be moderated. In this context, the iAs content of Hijiki and Hijiki-derived food products and/or supplements is being monitored for the period, 2016 to 2018, by several EU member states, as a request of the European Commission and EFSA [144]. By the end of that period, specific regulations will be set up in the case of any of the food products shows an iAs level that may pose a risk to human health. In addition, seaweeds have been increasingly included in animal feed since the predicted rising of the global population must certainly be accompanied of an increasing demand of livestock and livestock products. In this sense, studies aiming to understand the contribution of seaweed-enriched animal feeds to human metals intake has been increasing. Unlike the expected, recently, Monagail et al. [154] showed a low accumulation of total and inorganic As in humans by the consumption of livestock and livestock products fed with *Ascophyllum nodosum* enriched animal feed, probably due to detoxification steps and subsequent rapid excretion of As compounds from the bodies of livestock. Note, however, that *A. nodosum* is not recognised as a rich species in As compounds, and thus additional studies should be conducted with other seaweeds to increase the knowledge of these effects. However, it is also necessary to take into consideration that the understanding of a more realistic panorama related to the risks of iAs of macroalgae origin still demand the elucidation of its in vivo bioavailability.

Not less important, Cd, Hg, and Pb are also metals of major societal concern given their known undesirable effects on human health. According to WHO, Cd exposure may affect calcium metabolism, irreversible renal dysfunction as well as the development of lung cancer due to its carcinogenic effects [155]. In turn, Hg exposure may affect mainly central and peripheral nervous systems, resulting in several disorders at the nervous, digestive, and immune level, whose severity can depend on their route, duration of the exposure, and the toxicity of each type of Hg, i.e., elemental and methylmercury are more hazardous than ethylmercury [156]. Indeed, prenatal exposure through maternal consumption of foods containing methylmercury are currently of great concern since this has been associated with reduced performance on tests of neurologic function in children, including tests of cognitive development [157,158]. A meta-analysis performed by Axelrad et al. [159] considering data from three epidemiologic studies have shown a linear correlation between 1 µg/g increase in maternal hair mercury concentration and 0.18 decrease in infant intelligence quotient (IQ) tests. Instead, Pb exposure is not as harmful as Cd and Hg, although it can contribute to serious consequences at the brain and central nervous system, mainly of young children.

Data summarized in Table 5 suggests that brown macroalgae, particularly *Alaria esculenta*, can accumulate high levels of Cd (up to 8 mg/kg DW). In turn, maximum levels of Pb were found in a sample of *Gracilaria* spp. from Greece. Interestingly, Ródenas et al. [38] described that the amounts of Cd and Pb in European macroalgae samples were, on average, lower than those found in Korean and Japanese counterparts, possibly reflecting different levels of environmental pollution. Indeed, several studies focusing on pollution from many parts of the world, including China [153], Korea [153,163], Japan [64,153], India [164], Spain [49,73,153], and New Zealand [83], allow the suggestion that, regardless of the high range of variations of Cd and Pb levels in seaweeds, in general, those of Japan and Korea origins showed superior amounts of these contaminants [64,153,163]. Apart from that, it is relevant to remark that several analysed European macroalgae (Table 5) seem to exceed the French limits established for Cd (<0.5 mg/kg DW) and Pb (<5 mg/kg DW), while no limits were overcome for Hg (<0.1 mg/kg DW). Assuming the maximum values found for Cd and Pb in *A. esculenta* (Norway) and *Gracilaria* spp. (Greece), respectively, it is possible to conclude that the consumption of 8 g of that algae would provide 94% and 63% of the Cd and Pb TDI determined by WHO, respectively (0.068 mg Cd/day and 0.24 mg Pb/day for a body weight of 68 kg). Since the majority of the summarized European macroalgae exceed Cd and Pb allowed levels and contribute at a significant degree to TDIs, their consumption should be moderated. Consequently, it would be advisable to study the toxicological risks related to intake of these metals mainly on a population that follows a vegetarian lifestyle and macrobiotic diets that often include in their diet seaweeds and seaweed-derived food products.

Also of relevance, the reported data suggests that Sn levels are uniformly distributed among the three macroalgae phyla, although an exceptional value was found in a sample from *Laminaria* spp. from an unknown location. Instead, in general, red and brown seaweeds showed superior Sr contents comparatively to Chlorophyta, particularly *Laminaria* spp. The same predominance seems to occur for Al, for which levels in brown *A. esculenta* and *S. latissima* (both from Scotland) reached 8750 and 1877 mg/kg DW, respectively. Note that, excepting *Laminaria* spp., none of the European macroalgae represented in Table 5 exceed the French limits for Sn (<5 mg/kg DW).

## 7. Strengths and Limitations

In this work, a broad search of keywords that covered seaweeds’ elemental profile and content, health benefits and risks, consumption and food applications, and bioavailability was carried out, resulting in the compilation of data that was very dispersed until now. The search was conducted on multiple databases and was carried out by two independent authors. On the other hand, since no meta-analysis has been performed and data was subjectively presented, high susceptibility to reporting bias is a limitation of this work.

## 8. Concluding Remarks

The present review summarizes many pieces of information concerning the subject of minerals of macroalgae and applications in foods to present a broad perspective on this topic, which was quite disperse to date. Overall, studies conducted on macroalgaes of European origins showed a great ability of green macroalgae to accumulate Mg and Fe, whereas Mn and I are undoubtedly preferentially accumulated by Rhodophyta and Phaeophyta, offering them distinct potentialities as dietary mineral supplementation. Several authors also highlight that, the consumption of macroalgae could contribute to higher intakes of K, helping to balance the dietary Na/K ratio but on the other hand, it must be highlighted that levels of Na in macroalgae are in general high as well, and, if no salt replacement is considered, this fact may result in a global increment of Na intake in diets where this element is already above the recommended limits. Other worries also require some attention when considering the expansion of dietary consumption of macroalgae, namely, their high levels of iodine, particularly in Phaeophyta, and their capacity to accumulate significant levels of toxic metals. To answer these concerns, it would be opportune to establish specific regulations on European edible seaweeds. Still, further studies regarding the bioavailability of trace elements and toxic metals in vivo should be conducted to estimate the real concentrations of each element that are actually absorbed upon seaweed ingestion, as well as the possible health risks associated to the consumption of excessive amounts.

## Figures and Tables

**Table 1:** Macro- and trace elements content, expressed in g/kg and mg/kg DW, respectively, in some European edible seaweeds from different locations.

Species	Location	Macrominerals					Trace Elements								Reference
		Na	K	Ca	Mg	P	Fe	Mn	Cu	Zn	Co	Mo	Se	I	
**green macroalgae**															
*Ulva* spp.	Portugal	24	25	4–8	20–38	1.3	139–1100	13–94	3–33	16–64	0.2–1.4	0.3	0.2–2	23–114	[35,37,44]
	Spain	16–23	16–26	3.7–5.2	19–21	2	190–2830	16–91	2–9	4–30	0.1–0.6	-	0.3	8–66	[36,49,65,66,67,68]
	Italy	-	-	-	-	-	1033	-	13	64	-	-	-	-	[69]
	Ireland	11	12	20	24	3	353	86	22	17	0.3	-	<0.1	-	[70]
	Norway	-	-	4, 6	15–26	0.5–1	210–6000	11–130	5–6	8–25	-	-	0.03–0.1	21–130	[71]
	Unknown	-	12–26	2–5	-	3–7	-	32–637	3–23	6–35	-	-	-	28–64	[46,72]
**red macroalgae**															
*C. crispus*	Denmark	31	33	54	9	3	490	653	5	74	-	0.7	0.6	-	[52]
	Unknown	7–43	10–32	2–4	3–7	-	22–40	10–13	<5	9–71	0.1	0.1	-	-	[6,72,73]
*Gracilaria* spp.	Portugal	16	92	2	3–4	2	1049–2110	157–392	2–35	33	1.5	-	1	47	[35,44]
	Greece	-	-	-	-	-	95	-	2	95	-	-	-	-	[74]
	Denmark	10	49	4	3	1	352	502	2	24	-	0.2	-	-	[52]
*P. calcareum*	Unknown	-	1	303	-	0.6	-	175	14	15	7	-	-	34	[46]
*P. palmata*	Spain	3	44	7	2	-	114	233	4	15–46	0.4	-	-	77	[49,65,68]
	Norway	-	-	4	5	3	100	11	5	29	-	-	0.1	260	[71]
	Denmark	3	41	9	2	-	307	578	5	21	-	0.6	-	-	[52]
	Unknown	4	8–96	0.4–1	1	3	35	2–17	1–4	5–26	0.03	0.1	-	220	[73]
*Porphyra* spp.	Portugal	24	25–46	1–9	4–7	-	53–322	11–31	3–8	10–29	0.1–0.6	-	<0.2	-	[60,75]
	Spain	4–41	13–23	2–6	2–7	6	201–663	15–33	12–20	12–20	0.2–0.3	0.4	0.2–0.5	43	[36,38,45,49,65,68]
	France	44	24	7	8	1.5	149	23	10	82	0.3	1	0.5	-	[38,45]
	Unknown	2–36	7–35	1–4	4–6	5	103–156	27–37	3–14	14–74	0.1	0.2	-	42	[6,46,72,73]
**brown macroalgae**															
*A. esculenta*	Ireland	-	-	-	-	-	82–411	-	0.4–1	24–45	-	-	-	-	[76]
	Norway	12–61	20–32	7–31	4–12	2–3	52–850	2–22	1–4	7–49	0.2–1	0.4–3	0.04–1	220	[32,71,77]
	Scotland	-	-	-	-	-	180–1159	7–35	2–4	18–29	-	0.4–0.6	-	398–1238	[56]
*A. nodosum*	Spain	46–49	38–41	7–10	8.6–9	-	133–212	20–41	25	64	-	-	-	-	[34,78]
	Norway	31–52	14–87	11–14	8–10	0.5–2	150–370	10–16	2–9	28–96	3–5	0.1–1	<1	-	[32]
	Unknown	42	22–28	10–13	8	2	204	16–42	2–4	29	2	<1	<1	744–811	[46,79]
*F. spiralis*	Portugal	14	10–47	1–11	2–10	-	24–1205	14–98	1–2.4	15–740	0.2–2	-	<0.2	-	[60,75]
	Spain	39	40	7	9	-	448	145	45	71	-	-	-	-	[78]
*F. vesiculosus*	Portugal	23	41	14	8	-	88	547	30	30	-	-	-	-	[44]
	Spain	38	52	7	8	-	398	238	17	114	-	-	-	-	[78]
	UK	37	30	14	9	2	1500	-	80	-	-	-	-	700	[61]
	Norway	27–40	21–42	10–21	6–9	0.9–2	150–260	56–99	3–6	41–95	<3	<1	<1	-	[32,71]
	Unknown	55	24–43	9	10	1	42	55–72	5	37	-	-	-	655	[6,46]
*H. elongata*	Spain	18	78	12	2	-	14	10–47	2	4–40	0.4–1	-	-	117	[49,65,68]
	Unknown	5	8–62	3–5	3	1	4	7–49	1–4	6–49	0.2	0.03	-	122	[46,72,73]
*Laminaria spp.*	Spain	13–45	52–113	10–20	4–11	2	20–108	3–7	1–13	5–37	0.03–0.2	0.07	0.2–0.3	6138	[36,38,45,49,65,68]
	France	31	23	13	9	1	68	4	16	33	0.07	0.05	0.6	-	[38,45]
	Norway	-	-	10	8	2	13–58	1.6–4	0.6–2	6–24	0.03–0.3	-	0.02	3100	[71,77,80]
	Ireland	-	-	-	-	-	-	2–4	3–4	23–48	0.03–0.1	-	-	-	[81]
	Scotland	-	-	-	-	-	41–199	4–12	2–12	33–49	-	0.1–0.4	-	4761–9014	[56]
	Denmark	10	22	2	8	4	194	37	4	49	-	-	-	-	[57]
	Unknown	6–38	9–116	3–23	2–7	2	7–33	1–34	1–5	2–23	0.01	0.03	-	7316	[6,46,72,73]
*S. latissima*	Portugal	30	39	9–10	5–6	2	30–1854	4–6	1–38	39–42	0.4	-	1.3	958	[35,44]
	Norway	42–48	25–120	13–17	8–10	0.7–6	35–370	2–13	0.3–7	3–38	0.05–0.3	-	-	-	[32,77,80]
	Scotland	-	-	-	-		16–1159	7–45	2–5	8–31	-	0.1–1	-	39–4855	[56]
	Denmark	12	26	1	8	4	134	10	2	44	-	-	-	-	[82]
*U. pinnatifida*	Spain	52	63	13	10	-	43–46	1–7	1	4–22	0.1–0.4	-	0.5	306	[36,49,65,68]
	Unknown	7–71	9–87	3–17	3–12	3	9–170	1–19	1–7	3–136	0.03–0.4	0.03	-	191	[6,46,72,73]

**Table 2:** Contribution of macroalgae to recommended daily allowances of Na, K, Ca, Mg, and P.

Species	Na		K		Ca		Mg		P	
	Mean	Max.	Mean	Max.	Mean	Max.	Mean	Max.	Mean	Max.
**green macroalgae**										
*Ulva* spp.	7	10	8	10	6	20	49	80	3	7
**red macroalgae**										
*C. crispus*	11	17	9	13	18	54	14	20	3	-
*Gracilaria* spp.	5	6	28	37	3	4	7	9	2	3
*P. calcareum*	-	-	1	-	303	-	-	-	-	-
*P. palmata*	1.3	1.5	19	38	4	9	5	11	3	-
*Porphyra* spp.	10	17	10	19	4	7	11	17	5	6
**brown macroalgae**										
*A. esculenta*	15	24	10	13	15	31	17	26	3	4
*A. nodosum*	17	21	15	35	11	14	19	21	2	3
*F. spiralis*	11	16	13	19	7	11	15	21	-	-
*F. vesiculosus*	15	22	15	21	12	21	17	21	2	3
*H. elongata*	5	7	20	31	6	12	5	-	1	-
*Laminaria* spp.	10	18	22	46	11	23	15	24	3	4
*S. latissima*	13	19	21	48	10	17	16	20	4	6
*U. pinnatifida*	17	28	16	35	11	17	19	25	3	-

Values are expressed as %, considering a portion of 8 g of dry seaweed, and the mean and maximum values summarized in Table 1.

**Table 3:** Contribution of macroalgae to recommended daily allowances of Fe, Mn, Cu, Zn, Mo, Se, and I.

Species	Fe		Mn		Cu		Zn		Mo		Se		I	
	Mean	Max.	Mean	Max.	Mean	Max.	Mean	Max.	Mean	Max.	Mean	Max.	Mean	Max.
**green seaweeds**														
*Ulva* spp.	78	343	49	255	9	26	2	5	-	-	9	29	263	608
**red seaweeds**														
*C. crispus*	11	28	72	261	2.6	4	9	30	6	11	9	-	3221	-
*Gracilaria* spp.	37	121	92	201	6	28	7	13	3	-	15	-	251	-
*P. calcareum*	-	-	70	-	11	-	1.2	1	-	-	-	-	181	-
*P. palmata*	7	18	58	231	3	4	1.9	4	6	10	1.5	-	990	1387
*Porphyra* spp.	16	38	10	15	8.3	16	3	7	3.5	6.4	3.5	7.3	227	229
**brown seaweeds**														
*A. esculenta*	22	66	5	14	1.6	3	2.4	3.9	11	16	8	15	3300	6603
*A. nodosum*	12	21	10	17	15	56	4	8	11	16	8	15	5076	6933
*F. spiralis*	32	69	34	58	13	36	22	59	-	-	2.9		-	-
*F. vesiculosus*	19	86	63	219	15	64	4	9	-	-	8	15	2640	3733
*H. elongata*	0.5	0.8	8	20	2	3	1.7	3.2	0.5	-	-	-	637	651
*Laminaria* spp.	4	11	3	15	4	14	2.0	3.9	2	16	1.4	2.9	32,351	48,075
*S. latissima*	13	66	5	18	2	6	2.0	3.5	11	16	17	-	15,501	25,893
*U. pinnatifida*	4	10	4	8	3	6	4	11	-	-	7	-	1325	1632

Values are expressed as %, considering a portion of 8 g of dry seaweed, and the mean and maximum values summarized in Table 1.

**Table 4:** Studies reporting alterations in the mineral content of foods fortified with seaweeds.

Product	Seaweed Species	Relevant Results	Ref.
**meat and meat products**			
Frankfurters	*H. elongata* at 5%	↓ addition of NaCl to half	[96]
Beef patties	*U. pinnatifida* at 3%	↑ Na, K, Ca, and Mg; Na/K = 1	[97]
Meat emulsions	*H. elongata*, *U. pinnatifida*, or *P. umbilicalis* (2.5 or 5%)	↓ of NaCl addition from 2 to 0.5%	[98]
Restructured poultry	*H. elongata* (3%)	↓ of NaCl addition from 1.5 to 0.5%	[99]
Frankfurters	*H. elongata* (5.5%)	↓ of NaCl addition from 2 to 0.5%; ↑ K and Ca; Na/K < 1	[100]
Beef patties	*U. pinnatifida* (3%)	↑ Na, K, Ca, and Mg; Na/K = 1	[101]
Meat emulsions	*H. elongata*, *U. pinnatifida*, or *P. umbilicalis* (5.6%)	↓ Na and ↑ K, Ca, Mg, and Mn; ↑ Fe only in samples with *P. umbilicalis*; Na/K = 1	[102]
Pork	Feeding piglets with *A. nodosum* (10 or 20 g/kg feed)	↑ I content in piglet’s muscles and internal organs	[117]
Pork	Feeding pigs with *L. digitata* (1.2–1.9 g/kg feed)	↑ I content in pigs’ muscles and internal organs	[105]
**fish and fish products**			
Tuna jerkies	*Sargassum swartzii* (3 or 5%)	↑ Na, K, Ca, Mg, Mn, and Fe	[33]
Gilthead seabream	Feeding fish with *L. digitata* (10% in fish meal)	↑ I content in fish fillets	[109]
Rainbow trout	Feeding fish with *L. digitata* (3.65 g/kg fish meal)	↑ I content in fish fillets	[106]
**dairy products**			
Cottage cheese	*U. pinnatifida* (15%)	↑ Ca, Fe and Mg	[113]
Yogurt	*Laminaria* spp. (0.2 or 0.5%)	↑ I, Ca, K, Na, Mg, and Fe	[114]
Quark and fresh cheese	*S. latissima*	↑ I content	[115]
Milk	Feeding cattle with a mix of *U. rigida, Sargassum muticum,* and *Saccorhiza polyschides* (100 g/animal/day)	↑ I and Mo	[94]
**others**			
Eggs	Feeding laying hens with *Ulva prolifera* and *Cladophora* sp*.* enriched in Cu, Mn, Zn, Co, or Cr (1.8–48.3 g/50 kg feed)	↑ Cu, Mn, and Cr; ↑ K on eggs of hens fed with Cr-, Mn-, and Co-enriched algae; ↑ Ca in all eggs except those from hens fed with Cr- and Zn-enriched algae; ↑ Mg in all eggs except those from hens fed with Zn-enriched algae	[104]
Cookies	*Caulerpa racemosa* (1–10%)	↑ ash content	[16]
Egg noodles	*Monostroma nitidum* (3 or 6%)	↑ ash content	[118]
Hijiki mineral salt	*Sargassum* spp.	Hijiki salt was richer in K and other trace elements than conventional salts	[119]
*Ulva* mineral salt	*Ulva ohnoi* or *Ulva tepida*	↑ K, Ca, Mg, P, B, Cu, Mn, and Se; Na/K between 1.1 to 2.2	[120]

**Table 5:** Content of metalloids and toxic metals, expressed in mg/kg DW, in some edible European macroalgae, from different locations.

Species	Location	As	iAs	Cd	Pb	Hg	Sb	Sn	Sr	Al	Reference
**green macroalgae**											
*Ulva* spp.	Portugal	2–4	0.4	0.03–0.1	1–1.4	0.03					[37]
	Spain	0.8–3	-	0.1	1	-	<0.007	-	27–31	117	[36,49,68]
	Italy	7	-	0.2	7	-	-		-	-	[69]
	Ireland	6	-	0.3	1	<0.01	-	-	-	-	[70]
	Norway	5–8	-	0,1	-	0.005	-	-	-	-	[71]
	Unknown	2–15	0.2–0.4	0.03–4	0.5–7	0.02		1–2	27–63	<120	[46,72,153]
**red macroalgae**											
*C. crispus*	Unknown	4–26	0.2	0.3–1	0.1–5	0.006	-	3	83	8–120	[46,72,73]
*Gracilaria* spp.	Greece	-	-	0.8–3	10–19	-	-	-	-	-	[74]
	Italy	15	-	0.04–0.4	0.8–7	-	-	-	-	19–149	[69,160]
*P. palmata*	Spain	15	-	0.1–0.3	0.5	-	0.01	-	31	62	[49,68]
	Norway	10	-	0.5	-	0.005	-	-	-	-	[71]
	Denmark	8	0.3	<1	<1	<1	-	-	-	-	[86]
	Iceland	1	<0.03	<1	<1	-	-	-	-	-	[86]
	Unknown	8–10	0.4	0.2–0.7	0.05–4	0.01	-	1	3–71	32–120	[46,73,153]
*Porphyra* spp.	Portugal	-	-	0.4–1	0.1–0.2	<0.005	-	-	-	-	[60]
	Spain	9–19	0.1–0.6	0.1–3	0.3–0.5	0.008–0.03	0.01–0.02	-	2–130	15–890	[36,38,45,49,68]
	France	4	-	3	0.3	-	0.03	-	120	22	[38,45]
	Unknown	24–50	0.1–0.6	0.2–4	0.01–2	0.004–0.03	-	<1	25	<120	[46,72,153]
**brown macroalgae**											
*A. esculenta*	Ireland	<0.1	-	0.2–0.5	0.3–0.5	<0.07	-	0.2–1	-	-	[76]
	Norway	20–100	-	1–8	0.07–1	<0.005	<1	-	-	6–340	[32,77,80]
	Scotland	64–88	-	-	0.2–2	-	-	-	645–733	200–1877	[56]
*A. nodosum*	Norway	23–55	-	0.1–2	<0.5	<0.05	<1	-	-	49–210	[32]
	Unknown	37	-	1	3	-	-	5	598	<120	[46]
*F. spiralis*	Portugal	39	-	0.4–2	0.2–0.8	0.01	-	-	-	571	[60]
*F. vesiculosus*	Norway	36–59	-	0.9–3	<0.5	<0.05	<1	-	-	48–140	[32,71]
	Unknown	34–50	0.3	0.5–1	0.5–4	0.04	-	4	537	<120	[46,153]
*H. elongata*	Spain	8	-	-	0.3	-	0.01	-	58	7	[49,68]
	Unknown	33–37	0.2	0.3–0.8	0.02–3	0.008–0.02	-	<1	7–560	7–120	[46,72,73]
*S. fusiformis*	Unknown	99–147	32–85	1–3	0.5–0.9	0.01–0.05	-	-	-	-	[72,153,161]
*Laminaria* spp.	Spain	27–49	-	0.1–3.4	0.4–0.5	-	0.004–0.03	-	41–1980	10–91	[36,38,45,49,68]
	France	53	-	0.5	0.2	-	0.03	-	1270	40	[38,45]
	Norway	40–100	20	0.05–0.9	0.04–0.2	0.01–0.02	-	-	-	2–20	[71,77,80]
	Ireland	49–90	-	0.06–0.2	-	-	0.01–0.02	-	-	-	[81]
	Denmark	-	-	-	2	-	-	-	-	139	[82]
	Scotland	72–114	-	-	0.1–0.6	-	-	-	580–834	8–121	[56]
	Unknown	52–68	0.05–0.4	0.07–7	0.01–7	0.001–0.005	-	34	760	8–120	[46,72,73]
*S. latissima*	Portugal	67	-	1.6	0.2	0.1	-	-	-	11	[35]
	Norway	28–120	0.4	0.1–5	0.03–0.5	<0.05	<1	-	-	2–76	[32,77,80]
	Scotland	64–88	-	-	0.2–2	-	-	-	14–733	13–1877	[56]
	Denmark	-	-	-	1.5	-	-	-	-	107	[82]
*U. pinnatifida*	Spain	13–26	-	0.1–0.3	1	-	0.01	-	87	29	[36,49,68]
	Italy	20–70	-	0.1–0.2	2–7	-	-	-	1200	120	[69,162]
	Unknown	32–77	0.04–0.3	0.1–5	0.07–1	0.01–0.06	-	2	883	12–120	[46,72,73,153]
